# Role of Vascular Function in the Prognosis of Heart Failure Patients

**DOI:** 10.3390/jcm13092719

**Published:** 2024-05-06

**Authors:** Shunsuke Kiuchi, Shinji Hisatake, Shintaro Dobashi, Yoshiki Murakami, Takanori Ikeda

**Affiliations:** Department of Cardiovascular Medicine, Toho University Faculty of Medicine, Tokyo 143-8541, Japan

**Keywords:** blood pressure in-hospital variability, heart failure, prognosis, cardio–ankle brachial index, anemia

## Abstract

**Background:** Blood vessels have the Windkessel effect and are involved in blood circulation. The breakdown of this mechanism is also involved in the pathogenesis of heart failure (HF); however, the relationship between vascular dysfunction and HF prognosis is not fully understood. **Methods**: We evaluated 214 patients hospitalized for HF at our institution who underwent a cardio–ankle vascular index (CAVI), which evaluates vascular function, between January 2012 and July 2018. To investigate factors (including CAVI) associated with major adverse cardiac events (MACE) during 1 year after patients with HF were discharged, we evaluated clinical profiles, blood tests, chest X-P, 12-lead electrocardiography, and transthoracic echocardiographic findings. MACE was defined as cardiovascular death or readmission for HF. **Results**: The severity of HF between the MACE and non-MACE was not significantly different. Previous HF and chronic kidney disease were significantly more common in the MACE group. CAVI and % mean atrial pressure in the MACE group were statistically higher than those in the non-MACE group. The cardiac shadow as shown by chest X-P and left ventricular size in the MACE group were significantly bigger, and HF preserved ejection fraction (EF) (EF > 50%) was significantly more common in the MACE group. In multivariate analysis, CAVI was an independent predictive factor for the occurrence of MACE (model 1; hazard ratio (HR): 1.33, 95% confidence interval (CI): 1.05–1.68, *p* = 0.018; model 2; HR: 1.31, 95% CI: 1.07–1.60, *p* = 0.009). **Conclusions**: Because high CAVI is associated with poor prognosis of HF, these patients require more careful treatment.

## 1. Introduction

**Figure 1:**
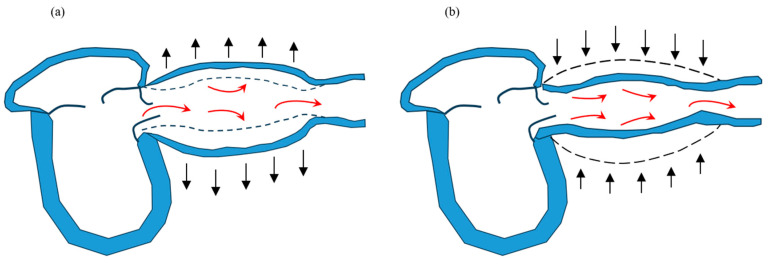
The Windkessel effect. During cardiac systole, approximately 60% of the blood ejected from the left ventricle pools in blood vessels (**a**), and during cardiac diastole, the pooled blood returns to the periphery (**b**).

In Japan, the number of patients with heart failure (HF) is estimated to increase by approximately 1.3 million by 2030 [1]. The United States has approximately 6.7 million patients with HF; thus, HF is a worldwide problem [2]. Additionally, HF has high mortality and re-hospitalization rates, resulting in a poor long-term prognosis. Although HF treatment advances yearly, the mortality and re-hospitalization rates have not decreased over time in Japan [3]. Therefore, early prognostic assessment and timely appropriate treatment are important. Previous studies reported that the number of hospitalizations for HF, total bilirubin (T-Bil), improved contractility, and other factors were associated with poor prognosis [4,5,6]. By contrast, vascular function is also important in HF. Usually, during left ventricular systole, approximately 40% of the blood ejected from the left ventricle is stored in the arteries, and the remaining 60% flows to the periphery [7]. During left ventricular diastole, the stored blood (40%) flows to the periphery. This mechanism is called the Windkessel effect, and vascular dysfunction contributes to HF by the breakdown of the Windkessel effect (Figure 1). When the Windkessel effect collapses, the increase in afterload caused by the elevated systolic blood pressure (BP) exacerbates HF. However, the relationship between vascular dysfunction and the prognosis of HF is not fully understood. Therefore, we evaluated how each factor, including vascular function assessment, affected the 1-year prognosis after patients with HR were discharged.

## 2. Materials and Methods

The present study was a single-center retrospective observational study in accordance with the Declaration of Helsinki (patients’ medical records were accessed for data collection). The study was approved by the Ethics Committee of Toho University Omori Medical Center (approval number: M21292_M18271_17318). The details of the study in opt-out format were posted on the website of our institution and our department (Department of Cardiovascular Medicine) and this acted as a waiver of informed consent from study participants. The subjects of the study were given the opportunity to decline to be enrolled.

### 2.1. Study Participants

**Figure 2:**
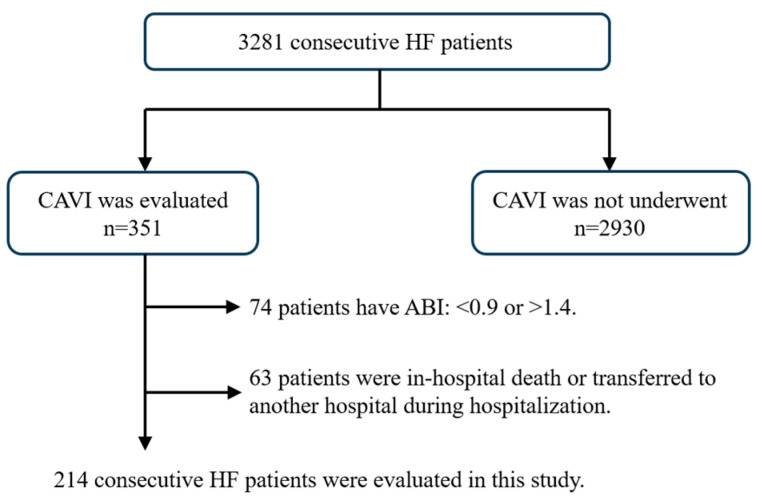
Selection of study populations. This flow chart illustrates the selection method of the present study’s participants. Overall, 214 consecutive heart failure patients were analyzed.

Figure 2 shows the subject flow chart of the present study. We evaluated 3281 consecutive patients hospitalized for HF at our institution between January 2012 and July 2018. HF was diagnosed based on the Framingham criteria or the guidelines of the American Heart Association or the European Society of Cardiology [8,9]. We investigated vascular function using CAVI and excluded patients whose ankle–brachial index (ABI) was <0.9 or >1.4. We also excluded patients with in-hospital death and those who transferred to another hospital and who could not be followed up for 1 year. The final cohort consisted of 214 patients.

### 2.2. Study Outcomes

The present study primarily aimed to investigate the factors (including CAVI) related to 1-year major adverse cardiac events (MACE) after patients with HF were discharged. MACE was defined as cardiovascular death or re-hospitalization with HF. We compared each factor (including CAVI) between the MACE and non-MACE groups. Multivariate analysis using the factors with statistically significant differences between the two groups was also performed. Furthermore, we created a receiver operating characteristic (ROC) curve of CAVI for predicting 1-year prognosis of HF, and a Kaplan–Meier curve was created using these values. Kaplan–Meier curves were also created for cardiovascular death or re-hospitalization with HF.

### 2.3. Patient Clinical Profiles

We investigated HF symptoms (New York Heart Association (NYHA) classification), length of hospital stay, and medications related to HF treatment at discharge. We evaluated the medications used for HF, such as beta-blockers (BBs), renin–angiotensin–aldosterone system inhibitors (RAS-Is), mineral corticoid receptor antagonists (MRAs), cardioprotective medications (BB, RAS-I, and MRA), and loop diuretics. Because the use of sodium glucose cotransporter (SGLT) 2 inhibitors had not been approved at the time of the study, we used triple therapy for cardioprotective therapy. Angiotensin-converting enzyme inhibitors or angiotensin II type 1a receptor blockers were considered as RAS-Is. Additionally, medical history and underlying heart disease were evaluated. We investigated hypertension (HT), diabetes mellitus, previous HF, and chronic kidney disease (CKD). Previous HF was defined as a history of previous hospitalization for HF. Underlying heart disease was classified into ischemic cardiomyopathy (ICM), hypertensive heart disease (HHD), valvular heart disease (VHD), tachycardia-induced cardiomyopathy (TIC), dilated cardiomyopathy (DCM), and others (including hypertrophic cardiomyopathy). Body mass index (BMI) was calculated using BMI = weight (kg)/height^2^ (m^2^), and we also evaluated physical features, such as age, sex, height, weight, and BMI.

### 2.4. BP and CAVI

We measured systolic and diastolic BP using an aneroid sphygmomanometer on admission and discharge [10]. The patients were divided into clinical scenario classifications (CS) of HF based on systolic BP at admission [11]. We calculated pulse pressure (PP) and mean BP using the following formula: PP = systolic BP − diastolic BP, mean BP, diastolic BP + PP/3. A nurse measured BPs during hospitalization three or more times (at 6:00, 10:00, 18:00, and other times). In-hospital BPV was evaluated using systolic BP (measured ≥ 8) for 3 days before discharge when the condition was stable. We calculated the coefficient of variation (CV) and standard deviation (SD) of systolic BP to evaluate in-hospital BP variability [12,13]. We defined CV as the within-patient SD divided by systolic BP. Heart rate (HR) was evaluated using standard 12-lead electrocardiography in the supine position on admission.

**Figure 3:**
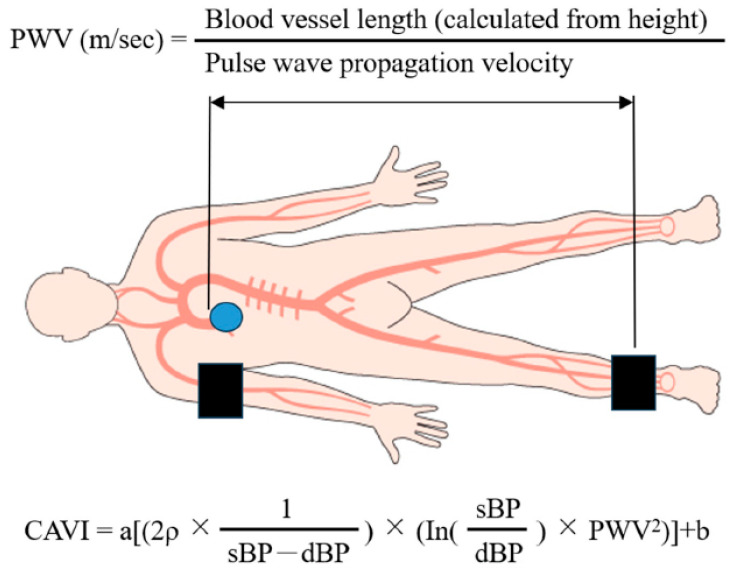
Graphic illustration of the CAVI measurement method.

We measured ABI/CAVI using a VaSera VS-1500E (Fukuda Denshi Company, Ltd., Tokyo, Japan) after 12 h of fasting in the morning following HF improvement, which was defined as a general condition suitable for discharge. Pulse wave velocity (PWV) was calculated using the time taken for the pulse wave to travel from the aortic valve to the ankle [14]. Using PWV, the CAVI was calculated as follows: CAVI = a[(2ρ × 1/(sBP − dBP)) × (In(sBP/dBP) × PWV2)] + b, where ρ is blood density, and a and b are constants to match aortic PWV (Figure 3). The right and left CAVI mean values were measured, as well as upstroke time and % mean atrial pressure (% MAP).

### 2.5. Other Clinical Examinations

Liver function (aspartate aminotransferase, alanine aminotransferase, and lactate dehydrogenase), renal function (JSNeGFR, blood urea nitrogen, and creatinine), hemoglobin (Hb), hematocrit, electrolytes (sodium and potassium), and brain natriuretic peptide (BNP) at admission were evaluated from laboratory examinations. Using the Japanese Society of Nephrology criteria, the JSNeGFR was calculated as JSNeGFR = 194 × Cr − 1.094 × age − 0.287 for men and 194 × Cr − 1.094 × age − 0.287 × 0.739 for women [15].

Two physicians blinded to the study evaluated the chest X-ray at admission to determine the cardiothoracic ratio (CTR). The CTR was calculated from the maximal cardiac and intrathoracic diameters.

We analyzed cardiac size, wall thickness, and left ventricle systolic function (ejection fraction: EF) from the transthoracic echocardiography performed by two physicians blinded to the present study. Cardiac size was measured using the left atrial dimension and left ventricular end-diastolic/-systolic dimensions, and interventricular septal and posterior left ventricular wall thicknesses at end-diastole were evaluated as wall thickness. We calculated the EF using either the modified Simpson method (apical two- or four-chamber view) or the Teichholz method (parasternal long-axis view) [16]. We also assessed the proportion of patients with HF reduced EF (HFrEF), HF mid-range EF (HFmrEF) and HF preserved EF (HFpEF). The Japan Circulation Society defines each type of HF as follows; HFrEF: EF ≦ 40%, HFmrEF: 40–50%, HFpEF: EF > 50%. [17].

### 2.6. Statistical Analysis

Data are presented as means ± SD. The unpaired Student’s *t*-test was used to compare the two groups. In all cases, differences with *p* value of <0.05 were considered statistically significant. We conducted multivariate analysis on factors that were found to be significant when compared between the two groups. Furthermore, the ROC curve was analyzed to determine an appropriate cutoff value of CAVI for predicting MACE. We used EZR (Saitama Medical Center, Jichi Medical University, Saitama, Japan), which is a graphical user interface for R (version 2.13.0, The R Foundation for Statistical Computing, Vienna, Austria) to conduct statistical analyses [18].

## 3. Results

### 3.1. Patient Backgrounds, Underlying Heart Disease, and HF Medications

Table 1 shows the patient characteristics of both groups. Compared with the non-MACE group, the MACE group was older, had a lower proportion of men, and had a smaller body size. Previous HF and CKD were also significantly more common in the MACE group.

In the non-MACE group, underlying heart diseases were ICM (37 patients, 23.6%), HHD (42 patients, 26.8%), VHD (37 patients, 23.6%), TIC (15 patients, 9.6%), DCM (16 patients, 10.2%), and others (10 patients, 6.4%). The MACE group diseases comprised ICM (12 patients, 21.1%), HHD (9 patients, 15.8%), VHD (18 patients, 31.6%), TIC (5 patients, 8.8%), DCM (7 patients, 12.3%), and others (6 patients, 10.5%). The MACE and non-MACE groups had several cases of VHD and HHD, respectively.

The introduction rate of cardioprotective medications, including BB, RAS-I, and MRA, as well as loop diuretics and their dosage, showed no differences.

### 3.2. Differences in HF Condition, and Cardiac and Renal Functions

HF severity using NYHA and BNP were not significantly different between the two groups (Table 1 and Table 2). The pathological condition of HF expressed with CS also showed no differences. Table 2 shows the results of blood examinations. BUN and JSNeGFR were significantly different between the two groups, with worse renal function in the MACE group. Although Hb showed no difference, hematocrit was significantly lower in the MACE group than in the non-MACE group.

In the non-MACE group, the cardiac shadow using chest X-P was significantly smaller (Table 2), and the size of the left ventricle using transthoracic echocardiography was also significantly smaller (Table 3). Left ventricular systolic function evaluated by EF was similar in both groups; however, HFpEF (EF > 50%) was significantly more common in the MACE group (Table 3).

### 3.3. BP Evaluation and CAVI

**Figure 4:**
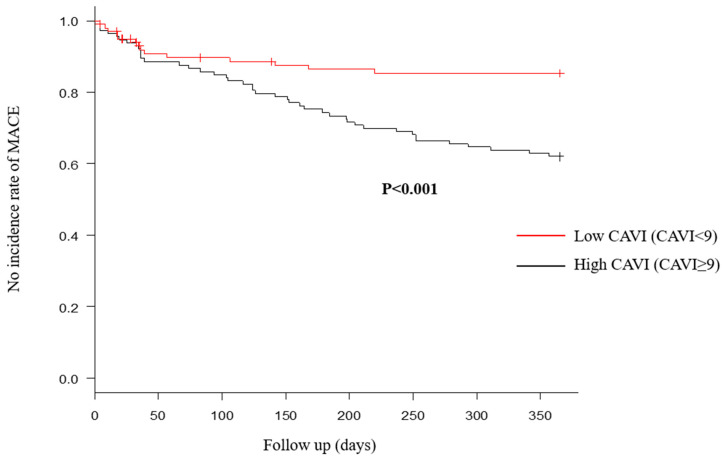
Major adverse cardiac events (MACE) within 1 year after discharge were compared between high and low cardio–ankle brachial index (CAVI) groups. Number of MACE within 1 year after discharge in the high CAVI group was significantly more, compared with the low CAVI group.

**Figure 5:**
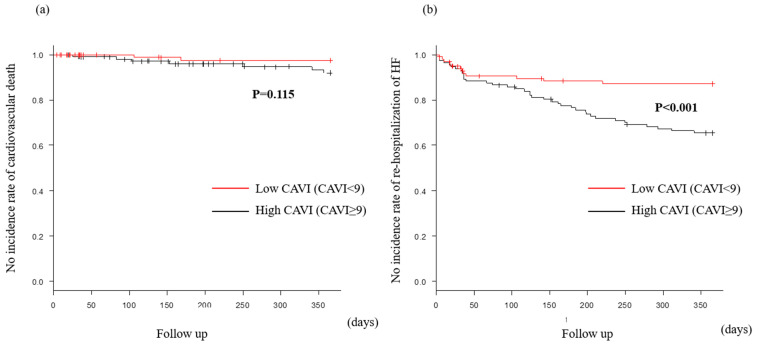
MACE within 1 year after discharge in the preserved CAVI group. Cardiovascular death (**a**) was similar in both groups. However, rehospitalization of heart failure (**b**) in the high CAVI group was significantly more, compared with the low CAVI group.

Table 4 shows ABI/CAVI results and BP assessment at admission. Systolic and diastolic BPs were not significantly different between the two groups, which was similar to the findings for mean BP and PP results. In addition, BP variability also showed no differences. By contrast, CAVI and % MAP were significantly different between the two groups. In particular, CAVI can independently predict MACE, even after adjusting for other factors (Table 5). The cutoff value of CAVI for predicting the composite endpoint was 9.0 from the ROC curve (sensitivity, 0.554; specificity, 0.754; area under the curve, 0.66; 95% confidence interval, 0.581–0.739). When comparing Kaplan−Meier curves between two groups using this value, the high CAVI group had significantly more MACE (Figure 4). Although re-hospitalization with HF was significantly different, cardiovascular mortality showed no significant difference (Figure 5).

## 4. Discussion

### 4.1. Main Findings

In the present study, previous HF, CKD, cardiac morphology (cardiac size and function), CAVI, and % MAP were associated with the occurrence of MACE. Most factors were reported as being associated with HF prognosis in previous studies. In particular, CAVI was an independent predictive factor for the occurrence of MACE. When divided into two groups based on CAVI, MACE occurred significantly more in the high CAVI group. In terms of MACE components, re-hospitalization with HF was significantly different, but cardiovascular death rates were not different.

### 4.2. Re-Hospitalization with HF and Prognosis

The greater the number of previous hospitalizations for HF, the worse the in-hospital mortality rate and poor prognosis 1 year after discharge [4,19]. In the past 9 years of longitudinal studies, the prognosis (mortality and/or re-hospitalization with HF) of patients with HF has not improved [3]. Re-hospitalization rates have not improved over time, and re-hospitalization with HF needs to be reduced first [20]. Factors leading to re-hospitalization with HF in Japan include increased salt intake, poor medication compliance, and arrhythmia. Approximately half of these factors can be addressed through patient self-management, and forms of self-management can reduce cardiovascular events by approximately 40% [21]. This study found that although all-cause mortality did not show differences, the risk of re-hospitalization with HF was reduced by approximately 50%. This result is consistent with the prediction that thorough self-management will reduce re-hospitalization with HF by approximately 50%. Although cardiovascular mortality was not different in the present study, re-hospitalization with HF was significantly reduced in the low CAVI group. The high CAVI group is a high-risk group for frequent re-hospitalization with HF, and stricter management, including thorough self-management, is important for these patients. Their prognosis could be improved by reducing re-hospitalization with HF.

### 4.3. Vascular Insufficiency and HF

HF is classified into HFrEF, HFmrEF, and HFpEF, based on left ventricular contractility [16]. Many treatments have been established for HFrEF, including cardioprotective medications, such as the fantastic four [22]. By contrast, the only cardioprotective treatment effective for HFpEF is sodium glucose cotransporter 2 (SGLT2) inhibitors, and medications are chosen based on individual pathological condition for HFmrEF. SGLT2 inhibitors are effective therapeutic medications for many pathological conditions, including reducing mortality in HF, recovered EF, and CKD [23,24]. Their mechanism may include effects on sympathetic nerve activity and cardiac energy metabolism [25,26]; however, this has not been established. As HFpEF includes various pathological conditions, only SGLT2 inhibitors with various actions are considered effective. The influence of vascular function contributes to the difference in treatment for the three types of HF (HFrEF, HFmrEF, and HFpEF) [27]. A decrease in the Windkessel effect causes an increase in systolic BP, which raises left ventricular afterload. This causes left ventricular hypertrophy, leading to left ventricular diastolic dysfunction by increasing cardiac work and oxygen demand [28]. Conversely, impaired coronary artery perfusion caused by a decrease in diastolic BP induces left ventricular systolic dysfunction due to decreased myocardial oxygen supply. Furthermore, vascular insufficiency results in coronary artery perfusion disorders via coronary arteriosclerosis. However, although some previous studies have reported an association between CAVI and HFpEF, which primarily involves diastolic dysfunction [29], there are few reports on all pathologies of HF. In the present study, we demonstrated that high CAVI was associated with a worsened prognosis in all cases of HF, and that vascular insufficiency is present in all pathologies of HF. Elevated PWV, which is used to calculate CAVI, was linked to prognosis in HFrEF without peripheral artery disease [30]. It has also been reported that CAVI can help predict hospitalization for worsening HF in patients with chronic HF [31]. Moreover, CAVI has also been linked to BNP [32], and BNP-related left ventricular filling pressure has been linked to CAVI [33]. Increased oxidative stress in failing hearts is linked to a poor prognosis [34], and CAVI is also thought to reflect oxidative stress [35]. Therefore, reduced oxidative stress could be responsible for these results. The current study was retrospective and oxidative stress and inflammatory markers could not be measured; therefore, a large-scale prospective study is required to confirm these hypotheses.

### 4.4. Evaluation of Vascular Function

Vascular insufficiency is a combination of vascular endothelial dysfunction, smooth muscle dysfunction, and metabolic dysfunction. Pathophysiological features range from asymptomatic early-stage vascular dysfunction to advanced atherosclerosis with some clinical symptoms. CAVI, PWV, flow-mediated vasodilation (FMD), and reactive hyperemia peripheral arterial tonometry (RH-PAT) are some of the methods used for evaluation [36]. FMD and RH-PAT can assess vascular endothelial function, whereas PWV can be roughly classified into two types based on the measurement site. This includes the carotid artery–femoral artery (cf) PWV and brachial–ankle (ba) PWV. The former mainly measures elastic arteries, and the latter focuses on muscular arteries. Heart–ankle (ha) PWV is used for CAVI measurement, but the target blood vessels are similar to baPWV. Like CAVI, haPWV has been linked to cardiovascular events [37], but it is heavily dependent on BP. However, unlike haPWV, CAVI is a BP independent index [14]. Although only CAVI was assessed in this retrospective study, there was no difference in BP between the MACE and non-MACE groups, and PWV may have produced similar results to CAVI. Furthermore, increased oxidative stress has been shown to cause vascular endothelial dysfunction, and a link between FMD or RH-PAT and oxidative stress has been reported [38,39]. Furthermore, it is believed that vascular endothelial damage may be linked to HF via oxidative stress, but this was not investigated in this retrospective study, and more research is needed.

### 4.5. Study Limitations

The present study was a single-center, retrospective study, which was limited by the small number of patients with HF whose vascular function was evaluated. The evaluation of CAVI in patients not suspected of having arteriosclerotic disease is not clinically useful, and it is also excluded from medical treatment covered by insurance. Therefore, we only evaluated the vascular function of patients who were suspected of having atherosclerotic disease. In the present study, the attending physician decided whether CAVI should be measured. Additionally, the extracted data may be limited because of the retrospective nature of the present study. Therefore, we were unable to evaluate patients’ quality of life, living environment, and nursing care. These potential limitations can undermine the strength of the study’s conclusions. Further clinical prospective studies in larger consecutive populations should be conducted to confirm our results.

## 5. Conclusions

As high CAVI is associated with poor prognosis of HF, these patients require more careful treatment.

## Figures and Tables

**Table 1:** Patient characteristics and medication at discharge in the MACE and non-MACE groups.

	Non-MACE Group (*n* = 157)	MACE Group (*n* = 57)	*p* Value
Age (years)	64.2 ± 13.9	70.9 ± 10.1	<0.001
Male (*n*, %)	115, 73.2	32, 56.1	0.017
Height (cm)	163.4 ± 9.8	159.0 ± 10.4	0.005
Weight (kg)	64.3 ± 17.4	54.3 ± 13.9	<0.001
Body mass index (kg/m^2^)	23.8 ± 4.9	21.4 ± 5.0	0.002
NYHA class (II/III/IV)	2/115/40	1/44/12	0.854
Hospital stay (days)	18.4 ± 11.0	21.1 ± 16.4	0.173
CS class (I/II/III)	83/67/7	28/25/4	0.494
Medical history of hypertension (*n*, %)	97, 61.8	43, 75.4	0.064
Medical history of diabetes (*n*, %)	44, 28.0	17, 29.8	0.798
Medical history of heart failure (*n*, %)	34, 21.7	23, 42.1	0.003
Medical history of CKD (*n*, %)	80, 51.0	47, 82.5	<0.001
Administration rate of β-blockers at discharge (*n*, %)	141, 89.7	51, 89.5	0.943
Administration rate of RAS-Is at discharge (*n*, %)	118, 75.2	42, 73.7	0.827
Administration rate of MRAs at discharge (*n*, %)	102, 65.0	31, 54.5	0.160
Administration rate of the above three medications at discharge (*n*, %)	71, 45.2	20, 35.1	0.187
Administration rate of loop diuretics at discharge (*n*, %)	103, 65.6	36, 63.2	0.427

MACE: major adverse cardiovascular events, NYHA: New York Heart Association, CS: clinical scenario, CKD: chronic kidney disease, RAS-I: renin–angiotensin–aldosterone system inhibitor, MRA: mineral corticoid receptor antagonist. Continuous data are expressed as the mean ± standard deviation. *p* values were determined using unpaired *t*-tests.

**Table 2:** Laboratory and chest X-P findings in the MACE and non-MACE groups.

	Non-MACE Group (*n* = 157)	MACE Group (*n* = 57)	*p* Value
Sodium (mg/dL)	139.2 ± 3.4	139.7 ± 3.3	0.407
Potassium (mg/dL)	4.1 ± 0.6	4.2 ± 0.6	0.258
AST (IU/L)	53.4 ± 87.0	76.6 ± 221.0	0.270
ALT (IU/L)	45.2 ± 87.0	64.5 ± 223.3	0.323
LDH (IU/L)	323.5 ± 132.6	353.7 ± 230.3	0.237
BUN (mg/dL)	19.8 ± 9.5	24.6 ± 14.2	0.005
Creatinine (mg/dL)	1.19 ± 1.44	1.35 ± 1.44	0.477
JSNeGFR (mL/min/1.73 m^2^)	60.1 ± 21.2	50.7 ± 23.3	0.006
White blood cell (/μL)	8697.3 ± 3228.0	7782.5 ± 3907.3	0.085
Hemoglobin (g/dL)	14.5 ± 9.8	12.3 ± 2.5	0.094
Hematocrit (%)	41.1 ± 5.8	37.2 ± 6.8	<0.001
Brain natriuretic peptide (pg/mL)	1089.9 ± 1188.1	1061.7 ± 706.3	0.866
Cardiothoracic ratio on admission (%)	60.8 ± 5.4	62.8 ± 5.3	0.016

MACE: major adverse cardiovascular events, AST: aspartate aminotransferase, ALT: alanine aminotransferase, LDH: lactate dehydrogenase, BUN: blood urea nitrogen, JSNeGFR: estimated glomerular filtration rate of the Japanese Society of Nephrology criteria. Continuous data are expressed as the mean ± standard deviation. *p* values were determined using unpaired *t*-tests.

**Table 3:** Transthoracic echocardiac findings in the MACE and non-MACE groups.

	Non-MACE Group (*n* = 157)	MACE Group (*n* = 57)	*p* Value
Left atrial dimension (mm)	43.6 ± 8.3	42.1 ± 7.9	0.238
Left ventricular end-diastolic dimension (mm)	59.6 ± 9.4	53.7 ± 9.0	<0.001
Left ventricular end-systolic dimension (mm)	46.5 ± 11.5	40.4 ± 11.6	<0.001
Interventricular septal thickness at end diastole (mm)	9.6 ± 2.5	10.0 ± 2.8	0.371
Posterior wall thickness at end diastole (mm)	10.1 ± 2.3	10.0 ± 2.5	0.818
Ejection fraction (%)	44.5 ± 16.0	48.4 ± 19.5	0.138
The proportion of HFrEF (*n*, %)	76, 48.4	22, 38.6	0.205
The proportion of HFmrEF (*n*, %)	29, 18.5	6, 10.5	0.166
The proportion of HFpEF (*n*, %)	52, 33.1	29, 50.9	0.018

MACE: major adverse cardiovascular events, HFrEF: heart failure reduced ejection fraction, HFmrEF: heart failure mid-range ejection fraction, HFpEF: heart failure preserved ejection fraction. Continuous data are expressed as the mean ± standard deviation or error. *p* values were determined using unpaired *t*-tests.

**Table 4:** CAVI and % MAP findings in the MACE and non-MACE groups.

	Non-MACE Group (*n* = 157)	MACE Group (*n* = 57)	*p* Value
Systolic blood pressure on admission (mmHg)	147.9 ± 32.9	145.1 ± 36.4	0.585
Diastolic blood pressure on admission (mmHg)	88.7 ± 23.9	82.6 ± 22.6	0.094
Mean blood pressure on admission (mmHg)	108.5 ± 25.3	103.4 ± 26.0	0.203
Pulse pressure on admission (mmHg)	59.2 ± 21.3	62.4 ± 21.7	0.327
Heart rate on admission (bpm)	100.5 ± 25.5	95.4 ± 24.4	0.196
Standard deviation	8.19 ± 2.88	8.55 ± 3.96	0.464
Coefficient of variation	7.29 ± 2.18	7.63 ± 2.91	0.351
Ankle–brachial index (right)	1.11 ± 0.11	1.11 ± 0.12	0.724
Ankle–brachial index (left)	1.10 ± 0.10	1.10 ± 0.12	0.772
CAVI (average)	8.51 ± 2.01	9.45 ± 1.34	0.001
Upstroke tine (right) (msec)	140.2 ± 26.4	140.6 ± 29.8	0.917
Upstroke tine (left) (msec)	137.9 ± 25.4	141.9 ± 32.7	0.351
% MAP (right) (%)	34.3 ± 6.2	36.9 ± 5.9	0.008
% MAP (left) (%)	33.9 ± 5.9	36.6 ± 7.0	0.005
% MAP (average) (%)	34.1 ± 5.6	36.8 ± 6.1	0.003

CAVI: cardio–ankle vascular index, % MAP: % mean atrial pressure, MACE: major adverse cardiovascular events. Continuous data are expressed as the mean ± standard deviation or error. *p* values were determined using unpaired *t*-tests.

**Table 5:** Multivariate analysis for prediction of MACE.

	HR	95% CI	*p* Value	HR	95% CI	*p* Value
Cardio–ankle vascular index	1.33	1.05–1.68	0.018	1.31	1.07–1.60	0.009
Heart failure with preserved ejection (%)	0.82	0.28–2.39	0.716			
Interventricular septal thickness at end diastole (mm)	1.89	0.49–7.38	0.358			
Left ventricular end-systolic dimension (mm)	0.97	0.92–1.01	0.164			
% mean atrial pressure (average) (%)	1.05	0.98–1.12	0.187			
Age (years)	1.01	0.98–1.05	0.498			
Hematocrit (%)				0.93	0.88–0.98	0.007
JSNeGFR (mL/min/1.73 m^2^)				0.99	0.97–1.01	0.188
Medical history of heart failure (*n*, %)				1.88	0.93–3.79	0.077
Cardiothoracic ratio on admission (%)				1.05	0.99–1.12	0.110

The multivariate analysis was performed applying the Cox proportional hazards models. MACE: major adverse cardiovascular events, HR: hazard ratio, CI: confidence interval. JSNeGFR: estimated glomerular filtration rate of the Japanese Society of Nephrology criteria.

## Data Availability

The datasets used and/or analyzed during the current study are available from the corresponding author upon reasonable request.
